# Chronic treatment with fluoxetine for more than 6 weeks decreases neurogenesis in the subventricular zone of adult mice

**DOI:** 10.1186/1756-6606-4-10

**Published:** 2011-03-08

**Authors:** Koji Ohira, Tsuyoshi Miyakawa

**Affiliations:** 1 Division of Systems Medical Science, Institute for Comprehensive Medical Science, Fujita Health University, Toyoake, Japan; 2 Core Research for Evolutional Science and Technology, Japan Science and Technology Agency, Kawaguchi, Japan; 3 Section of Behavior Patterns, Center for Genetic Analysis of Behavior, National Institute for Physiological Sciences, Okazaki, Japan

## Abstract

**Background:**

Recent studies indicate that chronic treatment with serotonergic antidepressants upregulates adult neurogenesis of the dentate gyrus (DG). In contrast, some studies claimed that there was very little alteration of neurogenesis in the subventricular zone (SVZ) by the antidepressants. Since almost all of those studies treated animals with drugs for 2 to 4 weeks as chronic treatment models of antidepressants, it is possible that antidepressant treatments for longer periods would affect adult neurogenesis in the SVZ.

**Results:**

In the present study, we examined the effects of long-term (up to 9 weeks) administration of fluoxetine (FLX), a selective serotonin reuptake inhibitor, on cell proliferation and survival in the DG and the SVZ of adult mice. As reported previously, in the DG of mice treated with FLX for 3, 6, or 9 weeks that were also injected with 5-bromodeoxyuridine (BrdU) in the last 3 days before perfusion, the numbers of Ki67- and BrdU-positive cells, which are cell proliferation markers, were significantly upregulated even at 3 weeks after the onset of the FLX treatments, and these increases were sustained in mice treated with FLX for 9 weeks. On the other hand, in the SVZ, we found a small, insignificant decrease in the numbers of Ki67- and BrdU-positive cells at 3 weeks, followed by highly significant decreases in the numbers of Ki67- and BrdU-positive cells at both 6 and 9 weeks. Furthermore, among olfactory newly generated cells that survived for 3 weeks after BrdU injection, the number of new cells was decreased at 9 weeks of FLX treatment.

**Conclusions:**

These results demonstrate that long-term (more than 6 weeks) treatment with FLX has the opposite effect on neurogenesis in the SVZ than it does in the DG. The results also suggest that the decrease in neurogenesis in the SVZ might be involved in some aspects of the drugs' therapeutic effects on depression. In addition, our findings raise the possibility that some of the side effects of antidepressants might be mediated by decreased adult neurogenesis in the SVZ.

## Background

It has been accepted that adult neurogenesis occurs in two regions, the hippocampal dentate gyrus (DG) and the anterior subventricular zone (SVZ), of the healthy adult mammalian brain throughout life [1]. Currently, a variety of factors that can modulate neurogenesis in these regions have been identified: drugs [2], exercise [3], environmental enrichment [4], pregnancy [5], and stroke upregulate neurogenesis [6], whereas stress [7] and aging [8] downregulate it.

Among the drugs that modulate adult neurogenesis, selective serotonin reuptake inhibitors (SSRIs) are the most-studied chemicals. Chronic treatment with SSRIs upregulates neurogenesis in the DG of the adult hippocampus [2,9], and this increase in neurogenesis seems to exert the antidepressant effects of SSRIs [9]. Increased extracellular serotonin (5-hydroxytryptamine, 5-HT) by SSRIs upregulates neurogenesis by increasing the proliferation of precursor cells [10] and cell survival [11]. 5-HT also gives rise to the upregulation of expression of neurotrophins, such as BDNF, which may stimulate differentiation and the survival of neurons [12]. Additionally, we have shown that fluoxetine (FLX), an SSRI, has the ability to alter the state of dentate granule cells. Chronic treatment with FLX can drastically reverse the established state of neuronal maturation in adult hippocampal granule cells [13], in a process called "dematuration", in which the cells display similar features to immature dentate gyrus of the mice heterozygous for the alpha-isoform of calcium/calmodulin-dependent protein kinase II in gene expression and electrophysiology [14]. It remains unclear whether or not dematuration of mature granule cells provides a therapeutic benefit for major depression and/or for side effects of FLX.

As described above, a considerable number of reports concerning the effects of FLX on hippocampal neurogenesis are available. In contrast, there are only a few reports on the effects of FLX on neurogenesis in the SVZ [2,9,10,15-18]. Almost all of the studies in the literature have revealed no influence of FLX on neurogenesis in the SVZ. In the experiments, the authors administered FLX for 2 to 4 weeks as a chronic treatment model, and the time courses that the authors used in the experiments on neurogenesis in the SVZ were the same as those in the DG [2,9,10,15-18]. Considering that 5-HT-containing fibers and 5-HT receptor subtypes can be detected in the SVZ [19] and that a pharmacological experiment with agonists and antagonists of 5-HT receptor subtypes suggested that 5-HT regulates neurogenesis in the SVZ [19], we hypothesized that FLX has a late-onset effect on neurogenesis in the SVZ. In the present study, to test this hypothesis, we administered FLX into adult mice for up to 9 weeks to examine whether or not FLX treatment affected neurogenesis in the SVZ.

## Results

### Chronic treatment with FLX has opposite effects on the regulation of cell proliferation in the DG than in the SVZ

**Figure 1:**
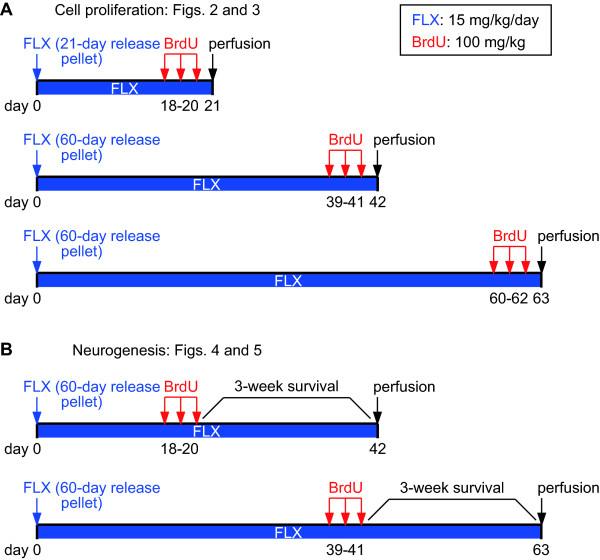
**Experimental designs**. (**A**) For cell proliferation studies, mice were killed 24 h after a single BrdU injection on each of the last 3 days in each administration period of FLX. (**B**) For neurogenesis studies, mice were killed 3 weeks after days of BrdU injections.

Cell proliferation in both the DG and SVZ was determined by immunohistochemical detection of Ki67, a nuclear protein expressed during all phases of the cell cycle, and 5-bromodeoxyuridine (BrdU), a thymidine analogue that is incorporated into DNA during the S-phase of the cell cycle. In the analysis of cell proliferation with BrdU treatment, mice were killed 24 h after a single BrdU injection on each of the last 3 days (Figure 1A) [20].

**Figure 2:**
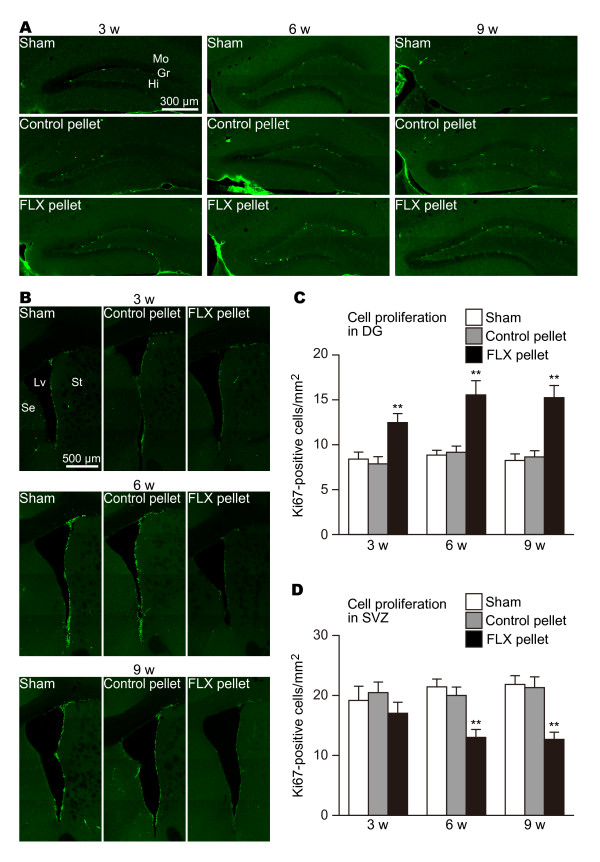
**Effects of FLX on the numbers of Ki67-positive cells in the DG and the SVZ**. (**A**) Cell proliferation in the DG. Ki67-positive structures, which are the nuclei of proliferating cells, stand in a line between the granule cell layer and the hilus. FLX treatments upregulated Ki67-positive cells in all periods, compared with sham-operated and control pellet-administered mice. (**B**) Cell proliferation in the SVZ. The numbers of Ki67-positive cells in FLX-treated mice were decreased at 6 and 9 weeks, but not at 3 weeks. The numbers of Ki67-positive cells in the DG (**C**) and the SVZ (**D**) are quantified. The values are means ± SEM of 4-5 animals in each group. ** p < 0.01 significantly different from the sham-operated group. Gr, granule cell layer; Hi, hilus; Lv, lateral ventricle; Mo, molecular layer; Se, septum; St, striatum.

**Figure 3:**
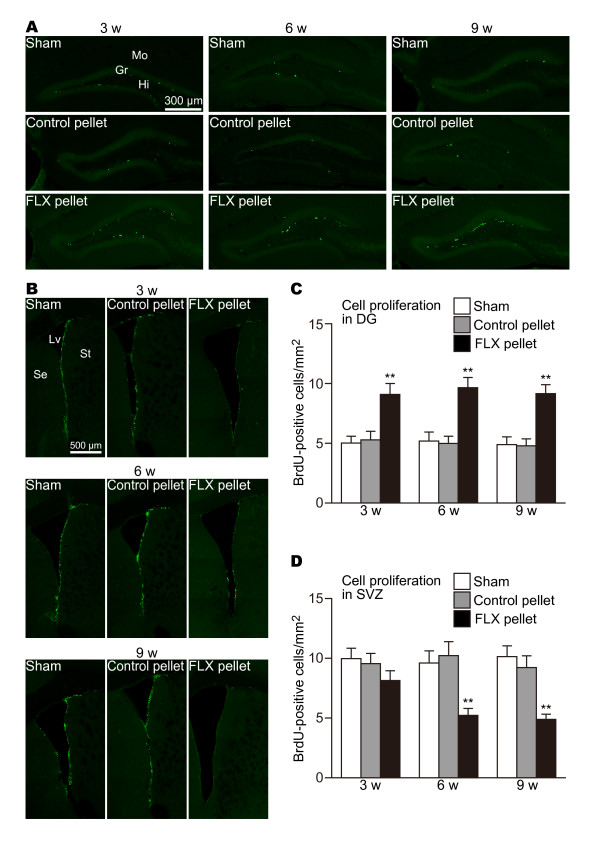
**Effects of FLX on the numbers of BrdU-positive cells in the DG and the SVZ**. (**A**) BrdU-positive structures, which are the nuclei of proliferating cells and early postmitotic immature granule cells, stand in a line between the granule cell layer and the hilus. FLX treatments upregulated BrdU-positive cells in all periods, compared with sham-operated and control pellet-administered mice. (**B**) BrdU-positive cells in the SVZ of FLX-treated mice. The numbers of BrdU-positive cells in the DG (**C**) and the SVZ (**D**) are quantified. The values are means ± SEM of 4-5 animals in each group. ** p < 0.01 significantly different from sham-operated group. Gr, granule cell layer; Hi, hilus; Lv, lateral ventricle; Mo, molecular layer; Se, septum; St, striatum.

In the DG, neural progenitor cells exist near the border between the hilus and the DG granule cell layer. Neuroblasts generated in the subgranular zone migrate radially a short distance into the granule cell layer and are integrated into the deepest portion of that layer, where they differentiate into granule cells. In this experiment, each mouse was given BrdU during the last 3 days before sacrifice. Since types 1 and 2a progenitor cells were labeled by BrdU [1], both Ki67-positive and BrdU-positive cells were observed around the subgranular zone. As shown in Figure 2A and 3A, almost all Ki67-positive and BrdU-positive cells were scattered in the subgranular zone and at the bottom of the granule cell layer. Significant increases in the numbers of Ki67-positive (Figure 2C; sham vs. FLX P = 0.00929, control vs. FLX P = 0.00491) and BrdU-positive cells (Figure 3C; sham vs. FLX P = 0.00386, control vs. FLX P = 0.00891) were already detected at 3 weeks. Increased numbers of Ki67-positive and BrdU-positive cells in the DG of mice treated with FLX rose slightly at 6 weeks (Ki67: sham vs. FLX, P = 0.00131, control vs. FLX P = 0.00233; BrdU: sham vs. FLX P = 0.00146, control vs. FLX P = 0.00051) and were sustained up to 9 weeks (Ki67: sham vs. FLX, P = 0.00083, control vs. FLX P = 0.00072; BrdU: sham vs. FLX P = 0.00073, control vs. FLX P = 0.00041). These results were well compatible with the obvious reports demonstrating that FLX's effect on the proliferation of neural progenitor cells appears by 3 weeks [2,10,11,15,17]. Such alterations of the numbers of Ki67-positive and BrdU-positive cells were not seen in the sham-operated or control pellet-administered mice during the experimental period.

In the SVZ, type-A cells (neuroblasts) are born throughout the SVZ, migrate in chains toward the olfactory bulb, and differentiate into granular or periglomerular interneurons [21]. The chains of type-A cells are ensheathed by type-B cells (SVZ GFAP-positive cells) [22,23]. Some type-B cells have been reported to work as neural stem cells. Type-C cells are clusters of rapidly dividing immature cells on the migration pathway physically located between type-B and type-A cells [23]. Thus, the SVZ neurogenic lineage is type-B cell (stem cell) > type-C cell (progenitor cell) > type-A cell (neuroblast). In this study, labeling of SVZ neurogenesis on the last 3 days before sacrifice can detect type-B and type-C cells located in the SVZ [1]. In addition, type-C cells migrate out from the SVZ. Thus, it is proven that BrdU-positive cells in the SVZ are almost certainly proliferating type-B and type-C cells. Actually, we found that both Ki67-positive (Figure 2B) and BrdU-positive cells (Figure 3B) were uniformly distributed in the SVZ of mice treated with FLX for 3 weeks. However, subtle though insignificant decreases in the numbers of Ki67-positive (Figure 2D; sham vs. FLX P = 0.494, control vs. FLX P = 0.207) and BrdU-positive cells (Figure 3D; sham vs. FLX P = 0.159, control vs. FLX P = 0.255) were observed. Interestingly, unlike the upregulation effect of FLX on cell proliferation in the DG, the treatments of FLX for more than 6 weeks significantly reduced the numbers of Ki67-positive (6 w: sham vs. FLX P = 0.00049, control vs. FLX P = 0.00255; 9 w: sham vs. FLX P = 0.00046, control vs. FLX P = 0.00189) and BrdU-positive cells (6 w: sham vs. FLX P = 0.00301, control vs. FLX P = 0.00179; 9 w: sham vs. FLX P = 0.00012, control vs. FLX P = 0.00111) in the SVZ, suggesting that the abilities of stem cells/progenitor cells to renew themselves and to produce daughter cells were downregulated by the FLX treatments. The downregulation of cell proliferation seemed to take place through the SVZ. Cell proliferation in the dorsal region between the corpus callosum and the striatum was reduced to a similar extent in the ventral region of the SVZ (Figure 2B, 3B).

### Chronic treatment with FLX has opposite effects on neurogenesis between the DG and the SVZ

The above data indicate that the FLX treatment has opposite effects on cell proliferation between the DG and the SVZ. Next, to determine whether or not the FLX treatment influenced neurogenesis in the DG and the SVZ, new cells were examined if they had survived a 3-week post-BrdU injection period during the FLX treatments for 6 and 9 weeks (Figure 1B). In this analysis, to detect new dentate granule cells, we used a double staining with BrdU and a neuronal marker, NeuN. We selected NeuN because the expression of NeuN is not affected by the FLX treatment [13]. In contrast, a mature granule cell marker, calbindin, has been shown to be greatly reduced by FLX administration [13].

**Figure 4:**
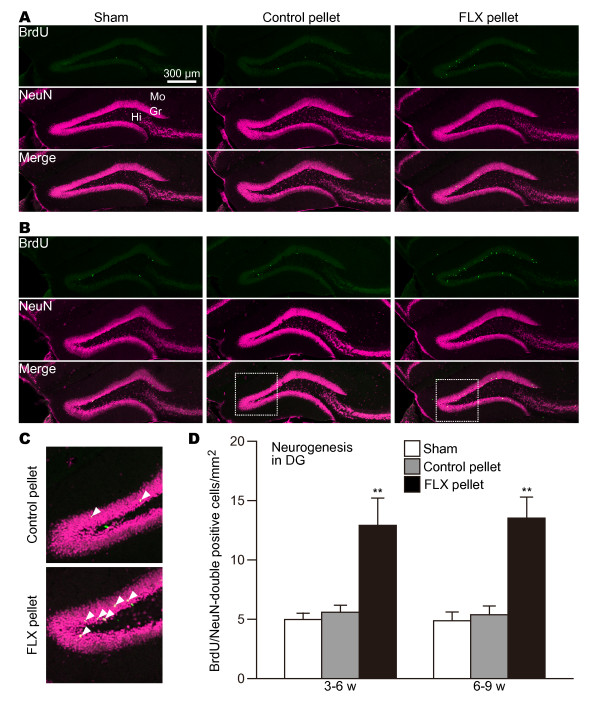
**Effects of FLX on the numbers of BrdU- and NeuN-positive cells in the DG**. (**A**) 3-week survival of new neurons in the DG of mice treated with FLX for 6 weeks. (**B**) 3-week survival of new neurons in the DG of mice treated with FLX for 9 weeks. Tissue sections in both **A **and **B **were double-stained with anti-BrdU (green) and anti-NeuN (magenta). The higher magnifications of the boxed-in areas in (**B**) are displayed in (**C**). (**C**) High-power images of BrdU and NeuN double-positive cells (arrowheads) in the DG in the control (top) and FLX-treated mice (bottom). (**D**) Quantification of BrdU and NeuN double-positive cells in the DG. The values are mean ± SEM of 4-5 animals in each group. ** p < 0.01 significantly different from sham-operated group. Gr, granule cell layer; Hi, hilus; Mo, molecular layer.

As expected from the above results, in the DG the number of BrdU/NeuN-double-positive cells was significantly increased by 6 and 9 weeks of chronic treatment compared with corresponding sham-operated and control groups (Figure 4; 3-6 w: sham vs. control P = 0.00468, control vs. FLX P = 0.00813; 6-9 w: sham vs. FLX P = 0.00050, control vs. FLX P = 0.00083). We found no difference in the numbers of BrdU/NeuN-double-positive cells between the 3-week period of 3-6 weeks and that of 6-9 weeks of FLX treatment (Figure 4D; 3-6 w vs. 6-9 w P = 0.837). This suggests that FLX's effect on neurogenesis in the DG reaches a plateau at 3 weeks into FLX treatment.

**Figure 5:**
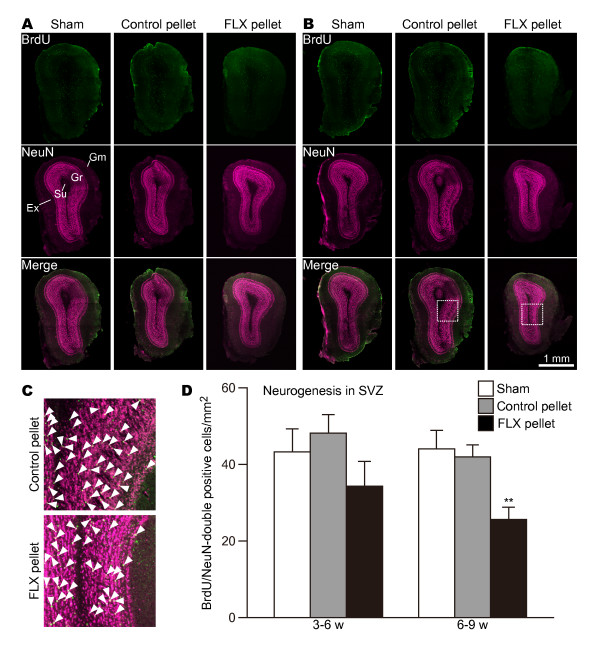
**Effects of FLX on the numbers of BrdU- and NeuN-positive cells in the olfactory bulb**. (**A**) 3-week survival of new neurons in the olfactory bulbs of mice treated with FLX for 6 weeks. (**B**) 3-week survival of new neurons in the olfactory bulbs of mice treated with FLX for 9 weeks. Tissue sections in both **A **and **B **were double-stained with anti-BrdU (green) and anti-NeuN (magenta). The higher magnifications of the boxed-in areas in (**B**) are displayed in (**C**). (**C**) High power images of BrdU and NeuN double-positive cells (arrowheads) in the olfactory bulbs in the control (top) and FLX-treated mice (bottom). (**D**) Quantification of BrdU and NeuN double-positive cells in the olfactory bulb. The values are means ± SEM of 4-5 animals in each group. ** p < 0.01 significantly different from sham-operated group. Ex, external plexiform layer; Gr, granule cell layer; Gm, glomerular layer; Su, subependymal zone.

In the SVZ-olfactory-bulb system, new neurons generated in the SVZ migrate to the olfactory bulb through the rostral migration stream and differentiate into granule cells or periglomerular cells [1]. To determine whether or not FLX treatment affected neurogenesis in the SVZ-olfactory-bulb system, we analyzed the alteration of the number of BrdU/NeuN-double-positive cells in the olfactory bulb. In the 3-week period of 3-6 weeks after FLX administration, we detected few significant changes in the numbers of BrdU/NeuN-double-positive cells in the olfactory bulb (Figure 5A, D; sham vs. FLX P = 0.328, control vs. FLX P = 0.108). In the analysis of mice that had been treated with FLX for 9 weeks and that had received 3-day injections of BrdU at 3 weeks before sacrifice, we found a significant reduction in the number of BrdU/NeuN-double-positive cells (Figure 5B-D; sham vs. FLX P = 0.00654, control vs. FLX P = 0.00264). This result demonstrates that FLX treatment decreases the number of new neurons in the olfactory bulb.

### FLX's effect on calbindin expression in dentate granule cells

**Figure 6:**
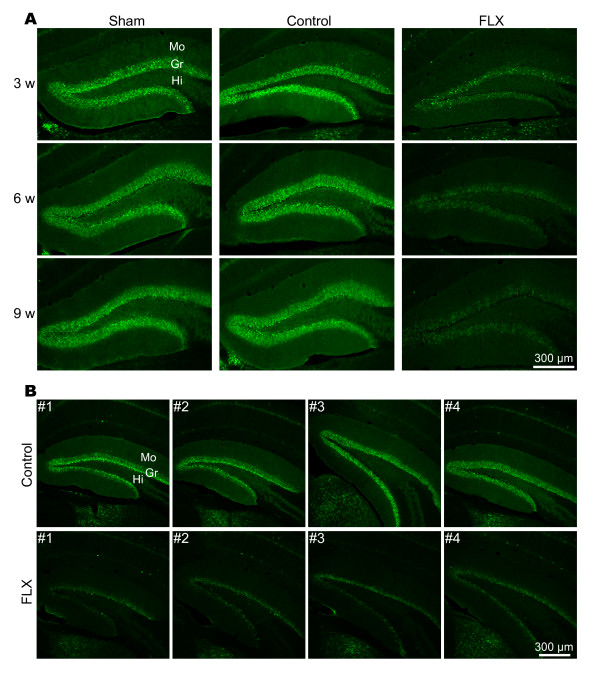
**Expression of calbindin in the DG of FLX-treated mice**. (**A**) Decreased expression of calbindin in FLX-treated mouse DG. Clear reductions in calbindin were found even at 3 weeks after FLX administration and sustained during FLX treatment. Control pellets did not affect calbindin expression, which was at a comparable level to the sham-operated mice. (**B**) Calbindin expression in the DG of mice treated with FLX for 6 weeks. Note that remarkable decreases in FLX expression were observed in all mice that had received FLX. Gr, granule cell layer; Hi, hilus; Mo, molecular layer.

As shown above, FLX's effect on neurogenesis in the SVZ was different from that in the DG depending on the duration of FLX treatment. Our group previously showed that chronic treatment with FLX reduces the expression of calbindin in the granule cells of the DG [13,14]. Thus, to examine whether or not FLX administration affects the dematuration of the DG depending on the duration of FLX treatment, we performed an immunohistochemical analysis of calbindin expression in the DG. Similarly depressed expressions of calbindin were clearly observed in the mice treated with FLX for 3, 6, and 9 weeks (Figure 6A). These decreases in calbindin expression were found in all mice treated with FLX for each duration (Figure 6B). These results suggest that FLX's effect on dematuration on the DG was unrelated to the duration of the treatment periods up to 9 weeks.

## Discussion

The present findings show that chronic treatment with FLX for more than 6 weeks can reduce cell proliferation in the SVZ, resulting in the downregulation of the number of new neurons in the olfactory bulb. In the DG, on the other hand, FLX treatment consistently increased cell proliferation and new neurons for up to 9 weeks. These findings suggest that long-term treatment with FLX, such as that lasting more than 6 weeks, has an opposite effect on cell proliferation in the SVZ than it does in the DG.

### Effects of chronic administration of FLX on cell proliferation and neurogenesis

Our data provided novel evidence that chronic FLX treatment for more than 6 weeks reduced cell proliferation and neurogenesis in the SVZ (Figure 2, 3, 5). The previous studies treated mice with FLX for time periods ranging from 1 day to 4 weeks, while we medicated mice with FLX for longer periods of up to 9 weeks. This prolonged period is critical for the reduction of cell proliferation and neurogenesis in the SVZ. Currently, the mechanism underlying late-onset reduction of cell proliferation and neurogenesis in the SVZ remains unclear. There are a few possibilities, as follows.

It could be postulated that the expressional changes of 5-HT receptors are a possible mechanism for the reduction of cell proliferation and neurogenesis in the SVZ during the treatment periods. To date, a number of 5-HT receptors have been identified, and some subtypes may be involved in adult neurogenesis in the SVZ, considering the evidence from pharmacological studies and the expression data of the receptors [24-28]. For example, treatment with ketansenin, a 5-HT2C receptor agonist, increases cell proliferation in the SVZ [19]. In contrast, treatment with sumatriptan, a 5-HT1B receptor agonist, decreases cell proliferation in the SVZ [19]. Thus, FLX-dependent altered and balanced expressions of the 5-HT receptors might become oriented toward the decrease in cell proliferation and neurogenesis in the SVZ. In light of cell proliferation and neurogenesis in the DG, the strong expression of the 5-HT1A receptor can be detected, and 8-hydroxy-2-(di-*n*-propylamino) tetralin, a 5-HT1A receptor agonist, elevates cell proliferation in the DG [11,19]. Since the expression level of the 5-HT1A receptor in the hippocampus cannot be altered by 3 weeks of FLX treatment [29], the stable enhancement of cell proliferation and neurogenesis may be detected in the DG. Thus, there is a difference in response to 5-HT between neural stem cells and progenitor cells in the DG and the SVZ, and the difference may be based on the expressions of 5-HT receptors.

Another possibility is the direct actions of FLX to nicotinic acetylcholine receptors (nAChRs). FLX has been shown to be an antagonist of nAChRs [30]. nAChRs play important roles in the enhancement of cell proliferation of neural stem cells and precursor cells [31]. Interestingly, the administration of nicotine increases cell proliferation in the SVZ of adult rats but not in the DG, and this effect is mediated by the induction of FGF-2 [32]. In contrast to the role of FGF-2 on cell proliferation in the SVZ, the apparent absence of an FGF-2 effect in the DG has been reported [33]. Thus, FLX treatments decrease FGF-2 expression via the inhibition of nAChRs, which may result in the reduction of cell proliferation and neurogenesis in the SVZ.

The possibility of mechanisms other than those described above cannot be excluded, and further study will be necessary to determine the mechanisms underlying the delayed reduction of cell proliferation neurogenesis in the SVZ.

### Implications of FLX treatment for depression

Citalopram, an SSRI, and clomipramine, a tricyclic antidepressant, induces a decrease in olfactory sensitivity after 3 weeks of treatment in mice [34]. The antidepressant rolipram, a monoamine oxidase inhibitor, is shown to impair the accuracy of mice in detecting odorants [35]. Considering that there is a positive correlation between the loss of olfactory function and reduced olfactory bulb volume [36,37], the loss of olfactory functions by treatment with antidepressants might be attributable to the reduction in new neurons in the olfactory bulb, which in turn is due to the decreases in both cell proliferation and neurogenesis in the SVZ. Also, some of the side effects of antidepressants might be mediated by decreased adult neurogenesis in the SVZ. A recent human case study showing that patients with major depression detect an unfavorable and intolerable smell about 7 weeks after the administration of citalopram may be related to the decrease in new neurons of the olfactory bulb [38]. In the animal models whose neurogenesis in the SVZ is ablated, it is found that the animals display a diminished behavioral fear response to conditioned odor cues [39], but their olfactory discrimination and long-term olfactory memory are not affected [39-41].

These data suggest that the decrease in neurogenesis in the SVZ might affect the emotional behaviors of animals via functional alterations of the circuits of the central nervous system as well as the olfactory bulb. The olfactory system forms a part of the limbic region that contributes to the emotional and memory components of animal behaviors. These areas, the frontal cortex-hippocampus-amygdala circuit, also seem to be dysfunctional in patients with major depression [42,43]. The olfactory bulb sends inhibitory projections to the amygdala, which is involved in the processing of emotion, such as fear, sadness, and aggression [44]. Dysfunction of the olfactory bulb might not only reduce olfactory sensitivity but also increase fear, sadness, and aggression by disinhibiting the amygdala [45,46]. Moreover, treatments with SSRIs seem to be a cause of aggression and violence as side effects [47]. The reduction of neurogenesis in the SVZ, which is shown in the present study, might be associated with certain side effects of SSRIs. Alternatively, the decrease in adult neurogenesis in the SVZ also might be involved in the therapeutic effects of SSRIs on major depression. The olfactory bulb-ablated animals have been reported to show markedly increased exploratory behaviors, such as ambulation and rearing [48], and spend significantly more time exploring a novel object into the center of the open field [49], suggesting that the reduced function of olfactory bulb may decrease the anxiety of the animals. Further researches are needed to clarify the functional and behavioural significances of the reduced SVZ adult neurogenesis caused by the chronic treatment of FLX.

There is a straightforward relationship between FLX treatment and the reduction of cell proliferation and neurogenesis in the SVZ of animals in healthy condition. However, there is a discrepancy in treatments with antidepressants to animal models of major depression. Bulbectomized rats, an animal model of major depression, have shown significant decreases in cell proliferation and neurogenesis in both the DG and the SVZ [50]. Contrary to the expectation that cell proliferation and neurogenesis would decrease by FLX treatment in depression model animals, treatment with imipramine, a tricyclic antidepressant, for 15 days normalized the reduction of neurogenesis in the SVZ in bulbectomized rats [50]. Furthermore, although chronic stress, which can be induce the onset of major depression, decreased the number of neural stem cells in the SVZ of adult mice, FLX treatment for 3 weeks reverses the decrease in the number of neural stem cells in the SVZ [51]. These reports suggest that quite opposite phenomena occur in depression model animals medicated with FLX compared with healthy control animals administered FLX. Although the mechanism that explains these discrepancies is unclear at present, we can speculate on two possibilities: 1) the alterations in the sensitivities of neural stem cells in the SVZ to 5-HT, containing functional and expressional changes of 5-HT receptors, transporters, and 5-HT signal transduction pathways; and 2) the direct influence of FLX on neural stem cells and progenitor cells, such as expressional changes of nAChRs in neural stem cells and progenitor cells. Such alterations might explain the different effects of antidepressants between control and model animals and also between major depression patients and healthy subjects.

## Conclusions

We have provided the first evidence for the the FLX-dependent decrease in adult neurogenesis in the SVZ. The reduction in neurogenesis in the SVZ by FLX treatment might be involved in some of the therapeutic effects on depression and side-effects of FLX, such as aggression and violence.

## Methods

### Antidepressant treatment

Adult male C57BL/6J mice (4-5 mice for each group and each time point; Charles River Laboratories, Japan, Inc., Shiga, Japan), which were 8 weeks old at the start of the experiments, were used for all of the experiments. All animal experiments were approved by the Institutional Animal Care and Use Committee of Fujita Health University, based on the Law for the Humane Treatment and Management of Animals (2005) and the Standards Relating to the Care and Management of Laboratory Animals and Relief of Pain (2006). Every effort was made to minimize the number of animals used. Animals were group-housed (12 h light/dark cycle) with free access to food and water. After 1 week of habituation to mouse cages, the mice were subcutaneously administered either an antidepressant or vehicle pellets (Innovative Research of America, Sarasota, FL) in the dorsal interscapular region of mice [52,53]. The drug pellets contained 7.245 mg and 20.7 mg of fluoxetine; these dosages were calculated so that a mouse with a body weight of 23 g received fluoxetine at 15 mg/kg/day for 21 days and 60 days, respectively (Figure 1). We have chosen the concentration of FLX, since serum FLX levels in mice receiving 15 mg/kg/day chronic FLX have been shown to be comparable to those in human patients receiving 20-80 mg FLX (Prozac) per day [54]. The pellets without fluoxetine were administered to mice designated as control mice. In addition, sham-operated mice, which were operated on but did not receive any pellets, were used.

Since the mice that we used were 8 weeks old at the start of the experiments, their body weight increased during the experiments. We then determined the actual dose of FLX corrected for body weight after 9 weeks of FLX treatment. The body weight in each group was as follows: sham-operated mice, 27.8 ± 0.317 g (n = 5); control pellet-administered mice, 27.3 ± 0.521 g (n = 5); FLX pellet-administrated mice, 26.7 ± 0.850 g (n = 5). Using these data, we calculated the actual dose of FLX as described below, 1000 g/[body weight (g) after 9 weeks of FLX treatment] × 0.345 mg (amount of FLX released from the 60-day pellet/day) = 13.0 ± 0.407 mg/kg/day. This value was within the plasma FLX levels for patients taking 20-80 mg Prozac per day [54].

### BrdU labeling

BrdU (Sigma, St. Louis, MO) stock solution was prepared in phosphate-buffered saline (PBS), (pH 7.2, 0.1 M) with 0.007 N NaOH at 20 mg/ml. After a certain period, the animals were injected intraperitoneally with BrdU (100 mg/kg body weight) every 24 h for 3 days to label newborn neurons (Figure 1).

### Immunohistological analysis

Mice were deeply anesthetized and transcardially perfused with 4% paraformaldehyde in 0.1 M phosphate buffer, pH 7.4. The brains were dissected, immersed overnight in the same fixative, and transferred to 30% sucrose in PBS for at least 3 days for cryoprotection. Brain samples were mounted in Tissue-Tek (Miles, Elkhart, IN), frozen, and cut into 50-μm-thick coronal sections using a microtome (CM1850, Leica Microsystems, Wetzlar, Germany). Sections were stored in PBS containing sodium azide (0.05%, w/v) at 4°C until use.

For BrdU staining, sections were incubated at 4°C for 10 min in 0.1 N HCl and then at 37°C for 30 min in 2 N HCl. Sections were washed twice for 5 min in PBS and then blocked in 0.2 M glycine in PBS at room temperature for at least 2 h. After washing in PBS for 1 h, all sections were preincubated with PBS-DB (4% normal donkey serum [Vector Laboratories, Burlingame, CA] and 1% BSA in PBS) for 2 h at room temperature. The sections were incubated at 4°C for 48 h or at room temperature overnight with the indicated primary antibodies. We used the following primary antibodies: mouse monoclonal anti-Ki-67 (1:20; BD Pharmingen, Franklin Lakes, NJ), mouse monoclonal anti-NeuN (1:200; Millipore, Billerica, MA), and rat monoclonal anti-BrdU (1:100; Abcam, Cambridge, UK). After washing in PBS for 1 h, the sections were incubated at room temperature for 1 h with the following secondary antibodies: anti-mouse IgG Alexa 488 (1:200; Molecular Probes, Eugene, OR), anti-rat IgG Alexa 594 (1:200; Molecular Probes). After washing in PBS containing Hoechst 33258 for nuclear counterstaining for 1 h, the sections were mounted on glass slides coated with 3-aminopropyltriethoxysilane and embedded with Permafluor (Thermo Shandon, Pittsburgh, PA). We used a confocal laser-scanning microscope (LSM 700; Carl Zeiss, Göttingen, Germany) to obtain images of the stained sections.

### Quantification of labeled cells

A quantification analysis was performed as reported previously [55]. Briefly, analysis was performed using a confocal microscope equipped with a 40× objective lens (Plan-NEOFLUAR, NA = 0.75, Carl Zeiss) and a pinhole setting that corresponded to a focal plane thickness of less than 1 μm. To exclude false-positives due to the overlay of signals from different cells, randomly selected positive cells were analyzed by moving through the entire z-axis of each cell. Cells were counted under the live mode of confocal scanning. Data were analyzed by one-way ANOVA and then by Scheffe's *post hoc *test. Error bars represent SEM.

## List of abbreviations

BrdU: 5-bromodeoxyuridine; DG: dentate gyrus; Ex; external plexiform layer; FLX: fluoxetine; Gm: glomerular layer; Gr: granule cell layer; Hi: hilus; 5-HT: 5-hydroxytryptamine; Lv: lateral ventricle; Mo: molecular layer; nAChR: nicotinic acetylcholine receptor; Se: septum; SSRI: selective serotonin reuptake inhibitor; St: striatum; Su: subependymal zone; SVZ: subventricular zone

## Competing interests

The authors declare that they have no competing interests.

## Authors' contributions

TM directed the study and wrote the manuscript. KO participated in the design of the study, carried out all the experiments, and co-wrote the manuscript. All authors read and approved the final manuscript.
